# Modeling the Efficacy of Oncolytic Adenoviruses In Vitro and In Vivo: Current and Future Perspectives

**DOI:** 10.3390/cancers12030619

**Published:** 2020-03-07

**Authors:** Mary K. McKenna, Amanda Rosewell-Shaw, Masataka Suzuki

**Affiliations:** 1 Baylor College of Medicine, Center for Cell Gene Therapy, Texas Children’s Hospital, Houston Methodist Hospital, Houston, TX 77030, USA; 2 Department of Medicine, Baylor College of Medicine, Houston, TX 77030, USA

**Keywords:** Oncolytic adenovirus, 3D modeling, in vivo modeling

## Abstract

Oncolytic adenoviruses (OAd) selectively target and lyse tumor cells and enhance anti- tumor immune responses. OAds have been used as promising cancer gene therapies for many years and there are a multitude of encouraging pre-clinical studies. However, translating OAd therapies to the clinic has had limited success, in part due to the lack of realistic pre-clinical models to rigorously test the efficacy of OAds. Solid tumors have a heterogenous and hostile microenvironment that provides many barriers to OAd treatment, including structural and immunosuppressive components that cannot be modeled in two-dimensional tissue culture. To replicate these characteristics and bridge the gap between pre-clinical and clinical success, studies must test OAd therapy in three-dimensional culture and animal models. This review focuses on current methods to test OAd efficacy in vitro and in vivo and the development of new model systems to test both oncolysis and immune stimulatory components of oncolytic adenovirotherapy.

## 1. Introduction

Human adenovirus (Ad) is one of the most studied viruses for cancer therapy and has shown great promise as a cancer treatment owing to its high gene transduction efficiency and genetic stability, as well as our ability to manufacture vectors to a high titer [1,2]. Ads are non-enveloped double stranded DNA viruses about 36 kb in size that are capable of infecting both replicating and non-replicating cells [3]. Oncolytic adenoviruses (OAds) can be easily manipulated to selectively replicate in cancer cells through modification of early replication transcriptional units, E1A and E1B, and by replacing the native E1 promoter with cancer cell specific promoters [4]. OAds selectively lyse tumor cells, disrupt the tumor microenvironment, and reactivate the immune system. Clinically, OAds have demonstrated a good safety profile and may address many of the most intractable obstacles to successful treatment of solid tumors [5].

Oncolysis of tumor cells after OAd infection releases pattern and damage-associated molecular patterns (PAMPs and DAMPs) and tumor-associated antigens (TAAs) that stimulate the immune response and enhance anti-tumor activity through epitope spreading or antigen cascade with limited clinical pathologies [6]. However, due to the heterogenous and complex nature of most solid tumors, OAd-mediated lysis is not sufficient to fully eliminate disease. Therefore, many studies have taken advantage of the well-characterized adenoviral vector to deliver immunostimulatory transgenes to the tumor site to further augment host immune activation, destroy stromal components, and prolong T-cell persistence [7,8,9].

Despite extensive research to optimize OAds as cancer immunotherapy agents, clinical responses have been modest [10]. One roadblock to predicting clinical efficacy from preclinical studies is identifying the correct model systems for OAd therapy. In order to model the biology of human OAd infection with the promotion of immunomodulatory effects, in vivo studies are required [11]. However, rodent cells do not permit complete human adenovirus replication, nor are xenograft tumors comprised of human stromal and immune components [12]. Researchers must also account for the additional cost of in vivo experiments, which are not practical for screening studies. Recent studies using 3D cultures and hydrogels to better recapitulate the physical tumor structure and microenvironment in vitro can be combined with advanced in vivo models, such as humanized mouse models, to elucidate the anti-tumor and anti-viral immune responses to OAd. This review will address current efforts to pre-clinically model the efficacy of oncolytic adenovirus against solid tumors and describe new approaches to bridge the gap between preclinical and clinical studies.

## 2. In Vitro Modeling of Oncolytic Adenovirus Therapy

### 2.1. Two-Dimensional Tissue Culture

Classical two-dimensional cell culture has been utilized since the early 1900s to study cell biology and has been adapted to many fields of research including oncology and virotherapy. This consists of a monolayer of single cells and is widely accepted due to excellent reproducibility in functional assays and ease of maintenance. Recent reviews comparing cell culture practices estimate that over 80% of current cancer research relies on conventional 2D cell culture because of its convenience, low cost, and ease of analysis [13]. The use of 2D culture to study oncolytic adenovirotherapy provides the means to test infectivity, viral replication and lysis, as well as permissiveness of noncancerous cells [11,14]. Yet, 2D culture has particular limitations when studying solid tumors and Ad infection as it fails to mimic the architecture of tumor, microenvironment, and non-cellular components such as the extracellular matrix (ECM) and intrinsic growth factors [15].

The general procedure to measure OAd infection in vitro consists of cells that primarily adhere to tissue culture treated plastic plates that are in contact with neighboring cells and media with viral particles applied at particular multiplicity of infection (MOI). This practice of infection does not reflect the necessity of OAds or other therapeutic molecules to cross multiple layers of cells to reach the intended targets [13]. Rather, a 2D monolayer results in an enlarged surface area of cells that may increase Ad receptor exposure for improved viral entry. A study comparing the expression of the receptor for the most commonly used Ad serotype 5 vectors, CAR (Coxsackie and adenovirus receptor), in normal mammary epithelial cells in 2D and 3D cultures showed that 2D culture of normal S1 cells had higher expression of CAR than in 3D models [16]. Additionally, altering the cellular microenvironment by culturing S1 cells in a collagen-based matrix re-polarized the cells and induced an upregulation of CAR expression compared to cells in 2D culture. This study suggests that culture conditions alter cell polarity and viral receptor expression and these factors should be accounted for when evaluating OAds in vitro.

Many solid tumors are of epithelial origin and when these cells are grown in 2D culture, polarization is limited with less than 5% of the cell membrane in contact with neighboring cells [17]. This loss of polarization in 2D may artificially promote increased OAd susceptibility. One study investigated the expression of different isoforms of CAR according to cell polarization in lung epithelia and Ad infection [18]. Two transmembrane isoforms, CAR^ex7^ and CAR^ex8^, reside on the basolateral surface and apical surface of polarized epithelia, respectively, and each mediates Ad entry from distinct epithelial surfaces [19]. However, the authors reported in non-polarized 2D cultured cells that both isoforms were accessible on the cell surface, resulting in three-log higher Ad transduction efficiency.

Further examination of 2D cultures suggests different patterns of gene expression emerge compared to in vivo and 3D culture experiments. In a study examining head and neck cancer cell lines, cancer stem cell (CSC) genes (*NANOG* and *SOX2*) were down regulated in 2D culture compared to 3D models [20]. Similar results were found in bladder cancer with T24 cell spheres expressing higher levels of mRNA and protein levels of NANOG compared to 2D culture [21]. These and other data suggest that 2D culture may reduce CSC populations, thus affecting the results of many functional assays. As CSCs are thought to be resistant to many treatment modalities and are the underlying cause of disease recurrence, their absence could artificially increase the efficacy of OAd treatment [22,23]. Encouragingly, oncolytic viruses are a promising treatment to target CSCs as OVs are able to utilize CSC proliferation machinery for replication [24], but must be modeled correctly to target this particular cancer cell population.

Many studies have utilized 2D cell culture methods to model OAd therapy and have led to successful preclinical in vivo models, yet cell line responses often do not predict human responses [25]. Due to the many limitations of 2D culture that fail to include components of the tumor microenvironment and may not accurately represent therapeutic efficacy, recent studies have investigated 3D preclinical models to better predict therapeutic outcomes. 

### 2.2. Three-Dimensional Tissue Culture

Solid tumors are heterogenous in cellularity, composed of malignant and noncancerous cells including endothelial cells, stromal cells, connective tissue, immune infiltrates, ECM (structural barriers), and other non-cellular components [26]. It has been shown that OAds have a limited ability to lyse tumor-associated stroma [9], which is an important factor to consider when measuring the overall efficacy of OAds. Thus, 3D models that allow investigators to incorporate some aspects of the solid tumor architecture may more closely mimic in vivo tumor complexity. Indeed, it has been shown that 3D spheroids are less susceptible to OAd spread compared to monolayer culture [11,13]. In addition, 3D culture systems may more closely simulate the growth kinetics of tumor lines in vivo [27].

There are many different 3D culture systems that generally can be grouped into three categories: multicellular tumor spheroids, hydrogels, and organotypic slice-based cultures (Table 1). Other 3D culture methods include bioscaffolds, bioreactors, and microfluidic systems, but these techniques have not been well utilized for OAd research and thus are beyond the scope of this review.

#### 2.2.1. Multicellular 3D Tumor Spheroids

3D tumor spheroids are aggregates of cells that can be grown in suspension on minimally adherent surfaces. Tumor spheroids have been reproducibly used for decades and can be composed of tumor cells only or can contain other cell types such as fibroblasts, mesenchymal stromal cells, and endothelial cells [36]. Depending on the source of cells (primary or cell line), tumors can form tight/dense spheroids, compact aggregates or loose aggregates depending on the cells’ adhesive properties or epithelial vs mesenchymal origin [37]. Tumor spheroids are generated through multiple methods including hanging drop, spinner flask, or liquid overlay of nonadherent surfaces such as agarose or polyHEMA [30], all of which produce spheroids that partially recapitulate the solid tumor microenvironment. These spheroids have a 3D architecture representative of cell-to-cell interactions, cell-produced ECM, proliferative exterior cells, necrotic cores (large spheroids > 1 mm diameter), as well as an enrichment for CSCs [13,15,22,38,39].

In a study comparing OAd infectivity of ovarian cancer cell lines in 2D monolayer vs 3D culture, Ad transgene expression as measured by luciferase signal was one to two logs higher in monolayer cultures [40]. However, this study also reported that cell line expression of CAR and integrins in both 2D and 3D models may not correspond well to primary tumors from patients. Later, the same group investigated the infectivity of five different OAds in 2D vs. 3D culture and confirmed that oncolysis was reduced in 3D compared to monolayer cultures [41]. Results also showed the chimera OAd5/3Δ24 (knob replaced to serotype 3: which utilizes DSG-2 as a receptor [42,43]) led to greater toxicity in 3D spheroids compared to wildtype CAR-utlizing OAd5, further supporting the difference in receptor and integrin expression in 3D spheroids compared to monolayer cutlure.

Testing OAd spread and replication in tumor spheroid models more closely mimics the in vivo setting compared to 2D monolayer cultures where the majority of cells are exposed to virus initially after infection [44]. One study showed that only about 23% of cells in a spheroid are exposed to the surface and thus an oxygen rich environment, while cells on the interior of the sphere have limited nutrients and low pH, as inside a solid tumor [40]. Correspondingly, much of the exterior cell population activily proliferates (G2/M phase) while interior cells are more quiescent (G0 phase) [45]. Thus, the organizatoin of these spheroids mimics many tumors which results in limited penetration of chemotherapeutics and oncolytic viruses in vivo. Studies utilizing primary glioblastoma spheroids showed Ad replication was required for viral spread to the interior of tumor, while replication defective Ads were restricted to the first layer of exterior cells [11]. Tumor spheroid models have also been used to study OAd in combination with radiation in glioma models [46]. Delivery of irradiation before OAd5Δ24RGD improved OAd penetration into the core of the spheroid and improved its anti-tumor efficacy compared to either therapy alone.

Some studies have generated tumor spheroids using ultra-low attachment (ULA) plates in order to maintain the CSC population, which is reduced in 2D culture [22]. In an A549 lung tumor model, scientists confirmed A549 sphere cells had stem-like properties (XIAP, Sox2, and NANOG expression) and found that an OAd expressing TRAIL (ZD55-TRAIL) targeted these CSCs and increased tumor cell apoptosis [47]. ULA plates used to culture three different liver cancer cell lines all showed increased expression of CSC markers (NANOG, Oct4, Sox2) and EMT markers [48]. Studies using an OAd regulated by GP73 promoter (GD55 OAd), a Golgi membrane glycoprotein elevated in hepatocellular carcinoma, sufficiently targeted CSCs in each of these three spheroid liver models. As many conventional therapies fail to eradicate CSCs, 3D models may be of particular value in evaluating the efficacy of OAds against this important cell population. A recent review discusses how CSCs escape cell death and the potential for oncolytic virotherapy to overcome these escape mechanisms [24], which could be tested in future 3D spheroid studies.

Overall, 3D spheroid cultures mimic the physical tumor microenvironment, including cell-to-cell interactions, but fail to fully recapitulate the ECM and growth factors present in solid tumors. While tumor spheroids have been generated with other cell types such as stromal and endothelial cells [36,49], studies investigating OAds in these models have yet to be completed. 

#### 2.2.2. 2D Hydrogels

Hydrogel systems may more closely mimic the interactions between tumor spheroids and the ECM. Collagen, fibronectin, elastin, and laminin make up the highly heterogenous and dynamic non-cellular structure of the tumor ECM, which is not only tissue specific, but also can be continually remodeled in vivo [50]. Hydrogels can be made of natural or synthetic ECM polymers such as Matrigel, collagen, or polyethylene glycol (PEG), but all provide a scaffold that maintains the biological phenotype of the cells and promotes nutrient exchange for long-term culture [44]. Hydrogels can also easily co-culture fibroblasts and endothelial cells with tumor cells [31]. In fact, some tumor models require the presence of stromal cells in a hydrogel system to form 3D structures. For example, in one study researchers mutated Ad5 to prevent binding to native Ad receptors and only enter through ανβ6 integrin, which is primarily overexpressed on pancreatic tumors [51]. The authors found that mutant Ad5-3Δ-A20T also infected pancreatic stellate cells, indicating improved viral spread within the microenvironment in this 3D hydrogel model that would have not been discernable in 2D culture.

To test how OAd therapy affects tumor-endothelial interactions, Jin and colleagues established a 3D prostate cancer model utilizing Matrigel as a scaffold system [52]. This system allowed co-culture of HUVEC cells with C4-2 prostate tumor cells to simulate angiogenesis in vitro, including the tubular formation of endothelial cells. The authors delivered a first-generation Ad expressing an antiangiogenic factor, Flk1, to block migration and formation of HUVEC cells within the hydrogel scaffold in combination with a replication competent Ad-hOC-E1, in which viral replication was driven by prostate cancer epithelial promoter. This dual Ad treatment inhibited tumor cell growth in vitro and induced complete regression in 30% of animals receiving both adenoviruses. Overall, this study supports the utility of targeting both the tumor itself and endothelial components of the microenvironment. Additional studies utilizing PEG-Fibrin based hydrogel systems have co-cultured lung adenocarcinoma cells with both lung derived fibroblasts and endothelial cells. These studies showed that delivering OAd with suicide genes improved anti-tumor activity in vitro and these results translated to effective tumor control in an orthotopic lung tumor model in vivo [31].

Hydrogel systems have also been used to develop, select, and isolate more effective OAds. Kuhn and colleagues tested the cytotoxicity of ovarian-targeting OAds derived from either a hydrogel or a traditional monolayer system [53]. Their “directed evolution” method allowed selection of a potent OAd after infection with an initial pool of different Ad serotypes. Ten serial passages were performed on 2D or Matrigel cultured ovarian cancer cells. Matrigel-derived OAd1 was more potent on multiple ovarian tumor cell lines and less cytotoxic to normal endothelial tissue than OAd2 derived from monolayer culture. This result was confirmed in vivo, with OAd1 controlling tumors better than OAd2 in an orthotopic ovarian tumor model. This study indicates that selection and isolation of OAds from similar therapeutic environments results in a more potent OAd that is already “primed” towards a solid tumor microenvironment.

To date, hydrogel systems are the best model to predict how OAd will interact with both tumor cells and components of the ECM, as these models provide structure as well as essential components of the tumor microenvironment [38]. As both the stroma and ECM inhibit viral spread in glioblastoma models, these models may be especially useful for testing the clinical efficacy of OAds against certain solid tumor types. Preclinical testing of OAd therapies in the presence of ECM will help predict the overall efficacy of Ad-mediated cytotoxicity in vivo. Still, there are some limitations of hydrogel systems to consider. For instance, some polymer scaffolds may limit viral spread, but this can be overcome by manipulating the size of the pores within hydrogels [54]. Additionally, hydrogel coating and cell seeding is more time intensive and expensive than most tumor spheroid models. There also may be difficulties in retrieving the cells from hydrogels after long-term culture, and current protocols involve enzymatic digestion (collagenase, nattokinase, hyaluronidase) that may reduce cell viability or dysregulate cell surface markers [54]. Due to these drawbacks, the OAd field has overall been reluctant to adopt this method for regular OAd evaluations. 

#### 2.2.3. 3D Organotypic Models

In vitro modeling using primary resected tumors most closely resembles in vivo tumors. Here, we will call these in vitro models organotypic slices/spheroids. Organotypic slices retain the overall architecture of the tumor with ECM components, stromal cells, endothelium, and immune infiltrates [55]. For this model, primary tumors are generally sliced to 250–300 μM thick and then placed in complete culture media or on top of hydrogels that contain collagen or laminin [29,38]. Freshly resected tumor sections can generally be cultured for 2–3 days with stable viability in a well oxygenated environment and also have the advantage of including healthy surrounding tissues [56]. In some cases, these slices spontaneously reorganize in suspension culture to form spheroids, though often they remain in resected form. These models are distinct from primary tumor spheroids, which are generally formed from single cell suspensions and lead to sphere formation, while organotypic spheroids are not dissociated into single cells before culture. Because these slices include the majority of the tumor microenvironment, they frequently are used to test the efficacy, repolarization of tumor immune microenvironment, and tumor-specific replication/spread of OAds.

Organotypic models were first used to test OAd specificity in ovarian cancer [57], and since have been used to determine the tumor specificity of many gene therapies. Screening a variety of breast cancer specific promoters in normal and malignant breast organotypic slices revealed a promising candidate in AdCXCR4 [58]. Subsequent studies show OAds under CXCR4 transcriptional targeting and fibroblast growth factor-2 (FGF2) translational control are significantly more specific for tumor tissue than normal breast tissue [59]. Breast organotypic slices have also been used to screen for infectivity of different OAd chimeric serotypes [60].

Studies investigating the effect of Ads expressing soluble TRAIL (AdsTRAIL) on glioblastoma organotypic slices found increased caspase 3 and TUNEL expression compared to slices treated with GFP expressing virus [61]. These results confirmed xenograft experiments and, importantly, allowed ex vivo testing on intact human samples. Thus, organotypic models are a means to tests OAd induced cell death on available human biopsies that predict and potentially replace in vivo animal models.

Systemic delivery of OAd therapy can cause liver damage and toxicity, especially with Ad5 serotypes, as hexon binding of blood coagulation factor X can result in liver sequestration [62,63]. Therefore, assessing the safety of OAd therapies and potential liver toxicities due to the inherent hepatotropism is essential prior to clinical translation. To test for the specificity of OAds, Rots et al. compared the tumor-on/liver-off profiles of wildtype Ad5 and a replication competent Ad with E1A under the control of a melanoma-specific tyrosine promoter that restricts replication to malignant melanoma cells (AdTyrE1) in healthy human liver organotypic slices [64]. While OAds were able to kill melanoma cells, E1 gene expression level of AdTyrE1was five-logs less than that of wild type Ad5 in human organotypic liver slices, suggesting a good tumor-on/liver-off ratio for AdTyrE1. This report suggests that ex vivo organotypic slices accurately reflect Ad activity in vivo and provide a rigorous in vitro model to test potential off-target replication.

Stoff-Khalili and colleagues also tested the cytotoxicity of OAdΔ24 compared to replication incompetent adenovirus on healthy liver slices from both humans and mice [65]. Despite reported limited replication of human Ads in mouse tissues, this study showed that OAdΔ24 was able to infect and induce apoptosis in both healthy human and mouse liver slices, albeit to a lesser extent in mice. Importantly, the authors compared the toxicity of OAdΔ24 and replication incompetent Ad (Adnull) in liver slices by release of aminotransferase enzymes as a marker for liver damage. The extracellular release of liver enzymes, including LDH, ALT, and AST were elevated in both human and mouse livers, suggesting that ex vivo organotypic livers are useful predictors of liver toxicity.

Organotypic cultures are one means to test how the 3D structural microenvironment affects OAd infection in vitro. But since these samples are derived from resected tumors, these models lack tumor vascularity and complete immune responses. Organotypic cultures do retain some immune infiltrate, however, which allows investigators to measure the effect of OAd on resident immune cells [56]. Still, these effects can only be tested in short-term cultures, as slices do not survive for longer than 4–5 days, which limits measurable OAd replication cycles. Further limiting the widespread use of this model, clinical access to tumor sections/biopsies is necessary and there is heterogeneity between samples, which makes studies difficult to reproduce. However, this drawback may in fact be of value given the development of personalized medicine, which can take advantage of individual patients slices to determine the safety and efficacy of OAd therapy before beginning full treatment regimens [38].

### 2.3. Oncolytic Adenovirus Studies in 3D Culture Models

As discussed above and summarized in Table 2, many different 3D culture models have been used to test the efficacy of OAds. Each model has benefits compared to 2D monolayer culture and allows for more rigorous pre-clinical testing to predict therapeutic efficacy in the clinical setting.

The majority of studies show that OAds lyse cells much less effectively in 3D compared to 2D cultures, suggesting that spatial organization regulates virus spread. A recent study comparing experimental 2D vs. 3D modeling to computational modeling to determine the efficacy of OAd spread confirmed this hypothesis [66]. Researchers found the average number of “neighbor” cells was higher in 3D models (median = 16) than 2D (median = 6), but virus spread was slower and less efficient, indicating that the additional dimension must be considered to accurately predict clinical efficacy. 

Another important factor to consider when testing different OAd vectors in 2D or 3D culture is the expression of CAR and other viral entry receptors. Both glioma and ovarian cancer models have shown that the availability of cell surface receptors in 3D models is more similar to primary tumors than in 2D models [11,41]. Normal breast epithelial S1 cells express high levels of CAR in 2D culture but minimal expression in 3D hydrogels, indicating that extracellular signals are required to maintain physiologic growth rate and CAR levels. Additionally, environmental factors and oxygen gradients are thought to affect the expression of CAR in both nonmalignant and tumor cells [16]. Hypoxia is thought to reduce replication efficiency and oncolysis in OAd therapy due to reduced E1A levels and hypoxia-induced G1 cell cycle arrest [67,68]. Large tumor spheroids generally have hypoxic cores which may prevent effective OAd spread and oncolysis but more closely mimic solid tumors in vivo [69]. 

While 3D culture models more closely resemble solid tumor structures, in vitro model systems still lack vascular components. As it provides a means for delivery and immune infiltration, angiogenesis plays an important role in tumor susceptibility to OAd. Angiogenesis is difficult to replicate in vitro and requires in vivo modeling. Additionally, the lack of immune components in 3D culture systems hinders the in vitro study of anti-viral and anti-tumor immune responses as well as targeting metastases through the abscopal effect [27,38]. Many OAd therapies require intratumoral injection for delivery due to the potential for recognition by cytotoxic T lymphocytes and antibody neutralization when delivered systemically. Currently, there are no in vitro models to recapitulate this delivery method due to size restrictions. Thus, animal models are required to correctly model the complete response to OAd therapy.

## 3. In Vivo Modeling of Oncolytic Adenoviruses

Similar to all oncolytic virotherapies, the goal of OAd treatment is to specifically lyse tumor cells, modulate the tumor microenvironment, release tumor antigens and DAMPs to stimulate host immune responses to cancer cells to improve overall anti-tumor activity [62]. As previously mentioned, many studies evaluating OAds have successfully shown targeted tumor cell lysis in pre-clinical models, both in vitro and in vivo, but have limited therapeutic efficacy in clinical trials. The immunology of Ad vectors in cancer gene therapy was recently reviewed and addresses how critical the anti-adenoviral and anti-tumor responses are for the efficacy of adenovirotherapies against cancer [73]. The field has made great progress in developing more relevant 3D in vitro models, but to fully test the efficacy and mechanisms of OAd therapy, in vivo models are required to measure the immune response to OAds. Here we will briefly cover historical and current in vivo models to evaluate OAds and discuss the potential of humanized mouse models that better predict clinical outcomes.

### 3.1. In Vivo Models of OAd Therapy

One major limitation for preclinical in vivo studies of OAds is the lack of permissive non-human hosts. Therefore, human xenografts in immunocompromised mice are the gold standard in vivo model to measure the therapeutic benefit of OAds [38]. Xenograft models provide an acceptable setting in which to evaluate OAd-mediated oncolysis; however, it remains impossible to measure anti-tumor and anti-viral immunity or the possible contribution of any viral-produced immunostimulatory molecules to OAd antitumor efficacy [7,74]. One study investigated the ability of wildtype Ad5 to replicate in seven different primary cell types: human, mouse, cotton rat, rabbit, swine, guinea pig, and woodchuck and determined that only porcine kidney cells replicated Ad5 at similar levels as human A549 lung cancer cells [12]. Still, there have been few follow-up studies using the porcine model for OAd therapies, possibly due to expense and lack of reagent availability. 

Cotton rat and Syrian hamster models are semi-permissive for human OAds. Both of these in vivo models are immunocompetent and involve respective syngeneic tumor cell lines allowing for human OAds to study their lytic effects, distributions, and generated immune responses as well as toxicities [75,76]. Recently, combination of OAd5Δ24 and infusion of ex vivo expanded tumor infiltrating lymphocytes induced systemic anti-tumor immunity in the Syrian hamster model [77]. Another study confirmed that T cells mediated anti-tumor activity in syrian hamsters and prior immunization with OAd resulted in clearance of the virus from tumors. Surprisingly, viral clearance did not dampen the T-cell anti-tumor response [78]. While these studies increase our understanding of T cell-mediated anti-viral and anti-tumor immunity, responses in hamsters may not translate well to the clinic due to differences in rodent and human immune systems. Also, the limited availability of cellular and antibody reagents for Syrian hamster and cotton rat models hinders further characterization of their immune responses and anti-tumor activity. Also, with limited syngeneic tumor lines, determining heterogenous responses against different tumor types in these models is difficult.

Due to the limited replication capacity of human OAds in other species, some groups have worked to identify murine tumor lines that are susceptible to OAd infection, such as a murine K-ras lung adenocarcinoma cell line, ADS-12 [79]. Infection of ADS-12 with AdTAV-255, an Ad vector dependent on tumor E1A and E1B expression, exhibited tumor control in 12954/SvJaeJ mice. The authors suggested that this immunocompetent model can be used to evaluate systemic immune responses to OAd therapy, and future studies should investigate this possibility. In a study testing the efficacy of human OAd D24-RGD against glioma in a C57Bl/6 mouse, results found increased immune infiltrates at the tumor only in the presence of the virus [80]. Encouragingly, results also showed increased IFNγ release from splenocytes after tumor stimulation, suggesting that D24-RGD stimulated a systemic immune response to reduce tumor burden and improve overall survival. In a syngeneic model of malignant mesothelioma in Balb/c mice, researchers showed an induction of T cells specific for Hexon, E1a, and mesothelin after multiple high dose intraperitoneal injections of ONCOS-102 suggesting an enhanced anti-tumor response [81]. Multiple injections of OAds can help partially compensate for limited replication observed in murine cells. Studies with immunocompetent mouse models require high dose OAd delivery that do not represent clinically relevant dosages. Other groups have worked to alter the OAd vector to specifically replicate in murine cells, but those studies have been difficult to reproduce. 

Currently, the majority of OAd in vivo testing remains in immunocompromised mice such as NSG (NOD *scid gamma*, or NOD.*Cg-Prkdc^scid^Il2rg^tm1Wjl^*/SzJ), athymic or nude mice with human xenografts. NSG mice lack B, T, and NK lymphocytes and have defective innate cells including macrophages and dendritic cells, while athymic mice only lack T cells. These mice allow for engraftment of many human tumor cell lines, as well as some primary human-derived xenografts (PDX). With the caveat of murine stromal components that may not fully recapitulate the human TME, xenograft and PDX models enable testing of how the microenvironment impacts OAd infection [82]. 

Studies testing the efficacy of OAds in xenograft models have yielded very promising results. For example, OAd DNX-2401 (or Delta24-RGD) targeting gliomas was original tested in athymic mice and led to an impressive median survival of 131 days compared to 19 days for untreated animals [83]. Reports from a recent phase I clinical trial have shown improved survival (>3 years) in 20% of patients receiving DNX-2401, with 3/25 patients showing greater than 95% reduction of tumor growth [84]. Studies investigating the efficacy of LOAd703, an OAd encoding CD40L and 4-1BBL costimulatory molecules, in pancreatic tumor models engrafted in C57Bl/6 nu/nu mice showed tumor control that was also observed with a LOAd (−) virus that did not express immunostimulatory transgenes [85]. The authors suggested that LOAd703 failed to improve tumor control compared to the transgene-negative virus due to the lack of T cells in this model. In order to validate the ability of LOAd703 to activate an immune response, the authors instead conducted in vitro experiments, which showed improved T and NK cell expansion. Based on these results, a clinical trial is currently underway to evaluate the efficacy and safety of LOAd703 in pancreatic cancer patients (NCT02705196).

Due to the lack of immune system in many of these animal models, these studies only truly test the effect of oncolysis in vivo and not the critical ability of OV to stimulate an immune response. Thus, in order to robustly evaluate their anti-tumor activity, OAd therapy must be tested in humanized mouse models with competent and complete immune systems [86].

### 3.2. Humanized Mouse Models for OAd Therapies

Recently, humanized mouse models have evolved to investigate the interactions of cell and gene therapies with cancer and the immune system. There are multiple humanized mouse models available, which are briefly summarized in Table 3 and more extensively reviewed elsewhere [87]. Generally, to create these models immunodeficient mice are injected with human cells, tissues, or stem cells that reconstitute in these animals a human immune system [88]. The development of any humanized mouse is complex, time consuming, and expensive compared to xenograft models, but they provide a unique environment to test OAd replication in human tumors and the ability to study human immune responses in vivo [73].

The simplest humanized mouse model is the Hu-PBL model where human peripheral blood mononuclear cells (PBMC) are injected into sub lethally irradiated adult NSG mice [89]. In this model, human T and B cells engraft within 2-4 weeks post injection. Studies have shown that with 2 × 10^7^ PBMCs injected, human CD45+ cells make up about 50% of Hu-PBL murine peripheral blood, with the majority being T cells [87]. Unfortunately, Hu-PBL mice develop xeno-GVHD, thus the model only offers a small therapeutic window. Other models include the engraftment of human CD34+ cells into either adult or fetal NSG mice [90]. For NSG-HSC mice, cord blood units are attained with RBC and T cell depletion. Both adult and fetal mice are sub lethally irradiated to deplete mouse HSCs and receive hCD34+ cells IV via different routes (adult: tail vein or retro-orbital; fetal: intrahepatic or intra-cardiac). The success rate of engraftment is much higher in fetal recipients compared to adults, but both take approximately 10 weeks to develop humanized cells [91]. In these animals, development of the human immune system is partially blocked as B cells retain an immature CD5+ phenotype and accumulate in the spleen, while T cells are restricted by HLA Class II and murine MHC [92]. The NSG-BLT model incorporates fetal liver and thymus transplants to provide a human thymic microenvironment for more human T cell development but may have increased risk for GVHD, as T cells that recognize mouse MHC can still develop [93]. To further optimize HLA-restricted T cell responses, an NSG-HLA-A2 mouse has been developed by transplanting Lin^−^CD34^+^CD38^−^ HSCs. This model allows both CD4+ and CD8+ T cells to generate Th1, Th2 and Th17 responses [94]. There is some variability in engraftment based on donor cells and the possibility of xenogenic GVHD in all of these models which may limit consistency and long-term monitoring, but humanized mice remain improved preclinical models to measure parts of the human immune response to OAd therapies.

Results from only a few studies in humanized mice have been reported to date. Kuryk and colleagues recently demonstrated that an Ad5/3 chimeric OAd armed with GMCSF (ONCOS-102) significantly reduced tumor volume when combined with pembrolizumab (anti-PD-L1) in huNOG mice (NOD/Shi-*scid*/IL-2Ry^null^) humanized with CD34+ cord blood cells [95]. ONCOS-102 had previously shown a good safety profile and tumor control in 40% of patients in a Phase I clinical trial (NCT01598129) [96]. As the investigators found increased CD8+ T cell infiltration at patient tumor sites, as well as increased PD-L1 expression, they hoped to combine ONCOS-102 with checkpoint inhibitors and thus utilized the huNOG mouse model for preclinical testing to approximate the clinical setting. With minimal toxicities reported in their humanized mouse model, this combination therapy warrants clinical testing.

## 4. Conclusions

**Figure 1:**
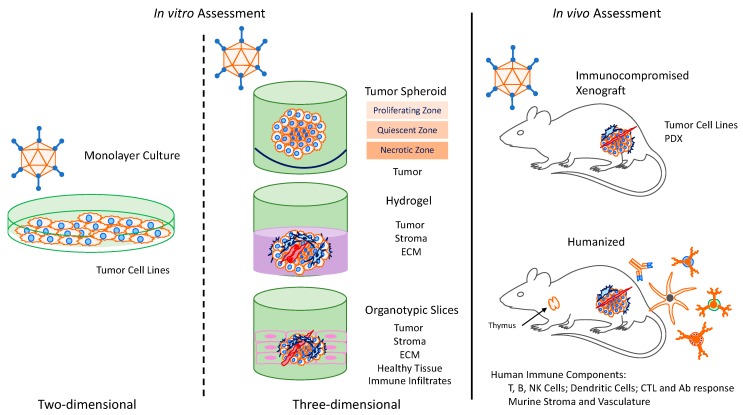
Preclinical models of oncolytic adenovirus.

Despite numerous encouraging pre-clinical reports of Oncolytic Adenoviruses to treat solid tumors, many of these studies have not translated to improved clinical outcomes. Here, we discuss how more accurate in vitro and in vivo models can evaluate the ability of OAds to not only induce oncolysis but also penetrate the solid tumor ECM and stimulate an immune response. Historically, 2D monolayer cultures have been used to provide a yes/no answer in determining the oncolytic effect of OAds against many tumors. We argue that these systems should continue to be used for routine screening of OAds of interest. However, monolayer culture cannot recapitulate the architecture of solid tumors, let alone provide the means to test the immune response post OAd infection. Thus, 3D culture systems provide a more realistic model in evaluating OAd virus spread, as well as infecting bystander cells with the inclusion of stromal and endothelial cells. Currently, 3D model systems are used as a secondary assay to validate traditional 2D culture, despite the fact that they more accurately model pre-clinical in vivo outcomes [27]. Still, it is difficult to simulate an immune response; one of the primary principles of oncolytic viroimmunotherapy in 3D models. Therefore, humanized mice are the best model in which to evaluate the overall human immune response to OAds (Figure 1).

Human xenograft mouse models are an excellent model to test oncolysis of many OAds but cannot predict the cascading immune response that follows OAd administration. Evolution to humanized mouse models may address this limitation and further close the gap between pre-clinical and clinical successes of OAd therapies. There are not many reports yet published using reliable humanized mouse models to test the effect of OAd therapy, but as development continues, we predict this model will more accurately predict patient responses. Many patients have immunological memory to Ad infection and future studies should utilize pre-immunized humanized mice to test how the memory response affects OAd therapy. These studies will enable more accurate prediction of therapeutic doses and OAd delivery systems. 

Currently, there is a shift in the OAd therapy field away from OAd monotherapy and toward combining OAd with immune checkpoint blockade or other cellular therapies including CAR-T cells [7,74]. Indeed, most clinical trials to date have shown that OAd alone is insufficient to eliminate both primary and metastatic tumors. With the addition of multiple immunotherapeutic platforms, 3D models will be invaluable in assessing therapeutic penetration (OAd, antibody therapy, cell therapy) in solid tumors. For these combination therapies, immunocompetent animal models that enable the evaluation of both oncolysis and human immune system will be especially important to address expected anti-tumor activity as well as mechanistic studies. More relevant preclinical models to test potential OAds will increase rigor in the field of oncolytic adenovirus therapy, reduce animal costs, and ultimately improve clinical outcomes.

## Figures and Tables

**Table 1:** Summary of current tissue culture systems.

Type of Model	Generation	Advantage/Characteristics	Limitation	References
2D culture	Tissue culture plate; monolayer adherent cell lines,	-Standard and accepted -Quick and inexpensive	-No solid tumor architecture -Lacks consistent predictable clinical outcomes	[20,28,29]
3D Tumor Spheroid	Ultra-low attachment surface-cells aggregate to form structure	-Relatively quick and simple -Mimic physical tumor structure -Outer cells proliferate Core is quiescent or necrotic (>1 mm diameter) -Promotes cancer stem cell (CSC) cells	-Avascular -Some cell lines require expensive coated plates or other biomaterials -Not all cell types generate spheroids	[30]
3D Hydrogels	Polyethylene glycol (PEG)/fibrin Matrigel Collagen	-Incorporates extracellular matrix (ECM) proteins -Provides scaffold and secreted factors for support to maintain phenotypes	-Gel systems may limit virus spread -Incorporation of other cell types is variable -Expensive and time requirements -Difficult to extract cells from gel	[27,31,32]
3D Organotypic Slices	Primary tumor (slices of resected tumor) OR growth on Bioscaffolds made of collagen or laminin	-Includes tumor and other stromal components and immune infiltrates from primary tumor -Sections allow for easier IHC staining	-Short term studies due to viability ex vivo -Low reproducibility -Scaffolds may affect adhesive properties -Limited Availability, IRB required	[33,34,35]

**Table 2:** Examples of 3D tissue modeling to test oncolytic adenoviruses.

3D Model	Generation	Tumor	Adenovirus	Summary	Reference
Spheroid	Agarose bed, spinning flask method	Glioma—U87 cells	Ad5Δ24RGD	Combined therapy with radiation. Radiation improved virus penetration to core of spheroid.	[46]
Spheroid	ULA plates with growth factors (EGF, bFGF and IGF1)	Lung—A549	ZD55-TRAIL Ad.DD3.D55	Oncolytic Ad targets CSC (cancer stem like cells) in spheroids. Induced apoptosis via mitochondrial pathway. OncAd improved survival in xenograft spheroids	[47]
Spheroid	ULA plate	Bladder	Onc.Ad5RGD	Targeting CIC (cancer initiating cells—like CSC) in bladder cancer T24 spheres. Spheres have low surface expression of CAR. RGD Ad increased improved infection	[21]
Spheroid	ULA; Spinner Flask method	Epithelial (T84—colon; A549—lung)	Ad3	Ads generate pento-dodechadera to increase lateral spread in epithelial tissues.	[70]
Spheroid	Agarose coated wells	Ovarian (SKOV.3)	Ad5 Ad5/3 Ad5Δ24RGD Ad5-Δ245/3	Compared spread of virus through luciferase activity between monolayer and 3D spheroid. Timing of infection is more representative in 3D. Ad5-Δ245/3 resulted in quicker lysis of ovarian tumor	[41]
Hydrogel	Matrigel	Prostate	Ad-Flk1-Fc	Ad virus delivery of Flk1-Fc fusion protein blocked VEGF receptor and reduced angiogenesis and tumor growth of prostate cancer cells and other stromal components. Combination of oncolytic and anti-angiogenic virus produced synergistic effect	[52]
Organotypic Spheroid	Agarose Coated wells	Glioblastoma	AdCMVLuc AdE1 + Luc	Non replicative Ad infected outer layer of spheroid. Replication competent Ad spread through spheroid. Used for intratumoral (intra-spheroid) spread studies CAR staining by IHC retained in spheroids derived from primary tumors	[11]
Organotypic spheroid	Primary ascites- organoid structure, suspension spheroids	Ovarian	Ad5 Ad5Δ24RGD	Spheroid improved viability of primary ovarian cancer lines Addition of RGD improved infection similar to monolayer culture	[40]
Organotypic spheroid	Hydrocell plates	Breast and glioma	AdΔE1B	Ad expressing decorin to degrade ECM and improve viral spread	[71]
Organotypic Spheroid	Xenograft model excised from mouse, digested and plated on agarose	Pancreatic	AdE1B19kDa	Onc.Ad expressing relaxin in combination with gemcitabine resulting in degradation of ECM	[72]

**Table 3:** Brief summary of humanized mouse models available.

Humanized Mouse Model	Human Cells	Advantages	Disadvantages	Reference
NOD SCID	Adult 34+ HSCs	Reconstitutes multiple hematopoietic lineages (T, B, NK, and myeloid: APCs)	40–70% BM chimerism of CD45+ cells Low consistency/engraftment	[91]
NSG-Hu-PBL	PBMC	Engraftment of T cells—presence of memory T cells Readily available source (healthy donors)	GVHD is common, utilized only for short-term study	[97]
NSG-HSC	Umbilical Cord Blood (CD34+ HSCs)	Improved engraftment to adult HSCs Reconstitutes hematopoietic lineages-generates naïve immune system	Engraftment dependent on donor. T cell educated by murine MHC	[90,97]
NSG-BLT	CD34+ HSCs and Fetal Human liver and thymus	Human lymphocytes Development of mucosal system	Other hematopoietic cells are chimeric (mouse/human)—minimum development 10 weeks Use of fetal liver and thymus	[93,98]
NSG-HLAA2	Lin-CD34+CD38- HSCs	Human HLA-A2-dependent/specific T cell development (T cell populations including αβ, γδ T cells and Th17 response) Functional CTLs	Lack of HLA-Class II	[94]
